# Protective Factors against Fear of Cancer Recurrence in Breast Cancer Patients: A Latent Growth Model

**DOI:** 10.3390/cancers15184590

**Published:** 2023-09-15

**Authors:** Gabriella Bentley, Osnat Zamir, Rawan Dahabre, Shlomit Perry, Evangelos C. Karademas, Paula Poikonen-Saksela, Ketti Mazzocco, Berta Sousa, Ruth Pat-Horenczyk

**Affiliations:** 1 School of Social Work and Social Welfare, Hebrew University of Jerusalem, Jerusalem 9190500, Israel; 2 Department of Psychology, University of Crete and Foundation for Research and Technology, 70013 Heraklion, Greece; 3 Helsinki University Hospital Comprehensive Cancer Center, University of Helsinki, 00100 Helsinki, Finland; 4 Department of Oncology and Hemato-Oncology, University of Milan, 20139 Milan, Italy; 5 Applied Research Division for Cognitive and Psychological Science, European Institute of Oncology IRCCS, 20139 Milan, Italy; 6 Breast Unit, Champalimaud Clinical Centre, Champalimaud Foundation, 1400-038 Lisboa, Portugal

**Keywords:** BOUNCE, breast cancer, coping self-efficacy, fear of cancer recurrence, latent growth modeling, trajectories

## Abstract

**Simple Summary:**

The present study’s objective was to examine the protective factors of fear of cancer recurrence (FCR) as well as its trajectory. The study encompassed a sample of 494 women participating in an international longitudinal research project named “Predicting Effective Adaptation to Breast Cancer to Help Women to BOUNCE Back” (BOUNCE). The participants had recently been diagnosed with breast cancer (BC), ranging from tumor stage I to III, and were undergoing BC treatments. The study underscores the stability observed in the FCR levels and highlights the influence of coping self-efficacy on the initial FCR levels. However, greater positive cognitive–emotion regulation did not appear to contribute to the level or reduction of FCR. These findings bear significant implications, emphasizing the necessity of targeted coping strategies for BC patients during a critical timeframe, to mitigate the impact of FCR, a factor that is liable to undermine the quality of life and mental well-being of BC survivors.

**Abstract:**

The current study aimed to examine the fear of cancer recurrence (FCR) trajectory and protective predictors in women coping with breast cancer (BC). The study’s model investigated whether a higher coping self-efficacy and positive cognitive–emotion regulation at the time of the BC diagnosis would lead to reduced levels of FCR at six months and in later stages (12 and 18 months) post-diagnosis. The sample included 494 women with stages I to III BC from Finland, Italy, Portugal, and Israel. They completed self-report questionnaires, including the Fear of Cancer Recurrence Inventory (FCRI-SF), the Cancer Behavior Inventory-Brief Version (CBI-B), the Cognitive–Emotion Regulation Questionnaire (CERQ short), and medical–social–demographic data. Findings revealed that a higher coping self-efficacy at diagnosis predicted lower FCR levels after six months but did not impact the FCR trajectory over time. Surprisingly, positive cognitive–emotion regulation did not predict FCR levels or changes over 18 months. FCR levels remained stable from six to 18 months post-diagnosis. This study emphasizes the importance of developing specific cancer coping skills, such as coping self-efficacy. Enhancing coping self-efficacy in the first six months after BC diagnosis may lead to lower FCR levels later, as FCR tends to persist in the following year.

## 1. Introduction

Breast cancer (BC) is the most prevalent type of cancer diagnosed among women [1] and involves a range of physical and psychological long-term issues, one of which is fear of cancer recurrence (FCR). FCR refers to the “worry or concern relating to the possibility that cancer will come back or progress” [2] (p. 3265). Such fears have been recognized as the most common concern of cancer patients [3,4,5] and as a central unmet need of women coping with BC [6]. Breast cancer patients often undergo significant changes in their identity and physical appearance, such as scars, fluctuations in weight, and other treatment-related side effects, which may be perceived as sources of danger and fear that may develop into FCR [7,8]. FCR manifests through cancer-related thoughts and feelings, such as about death, loneliness, and uncertainty [9], and it is likely to impair the quality of life and mental health [10,11]. 

FCR is present through the time of active disease, after completion of treatment [12], and along the survivorship trajectory [12,13,14]. Even years after the initial diagnosis, most survivors will still feel stressed about cancer progression or recurrence [13]. As such, for many cancer patients, surviving cancer means living with ongoing apprehension of the cancer recurring [12]. Several studies have indicated that FCR levels typically exhibit a relatively stable pattern throughout the survivorship cycle. For example, the initial FCR levels in BC patients obtained on the first day of radiation treatment were found as a strong predictor for the levels of FCR 2 months later [15] and throughout 18 months after completing treatment [16]. In addition, while some studies indicated that clinical levels of FCR at baseline tend to remain stable even 18 months after surgery [17,18], other studies have shown that FCR levels may be higher before surgery, but they tend to mildly decline, and then plateau [19,20]. Significant changes in FCR occurred during the month of pre-mammogram to the month post-mammogram assessment in another study [21]. Thus, the inconsistent evidence regarding the FCR trajectory among BC patients demands further examination of this issue. 

While extensive research shows that FCR is a constant difficulty in the lives of cancer survivors, not much is known about protective factors against FCR. Previous studies have presented factors correlated with FCR, such as psychological distress, intrusive thoughts [11], female gender, and younger age [22]. A recent meta-analysis of a large heterogeneous cancer sample (N = 13,000) aimed to detect factors correlated with FCR, such as anxiety, depression, chemotherapy, and fatigue, which were positively associated with FCR, whereas optimism, social support, and quality of life were negatively associated with FCR [23]. Nevertheless, most of these studies showed correlations with FCR, but it is difficult to infer from them a protective and moderating role against FCR. For example, the study by McGinty et al. [21] found that reporting lower perceived risk and reassuring behaviors in BC patients had a protective role in FCR. Considering the increasing and relatively high survival rate of BC [24,25], it is imperative to identify initial protective factors in BC patients, especially at the crucial time of diagnosis, that can protect against FCR and its progression during cancer treatment and survival.

The Common-Sense Model (CSM) [26,27] postulated that coping with illness depends on two central representations of the illness, cognitive and emotional. The cognitive representation refers to the perceived health threat [27,28], including the evaluation of the duration and chronicity of the disease, the consequences of cancer, the perceived control, and the potential for a cure. These perceptions depend on the individual’s history, knowledge, and beliefs about the illness. The perceived health threat may further induce emotional representations [26,27]. For instance, it may provoke worry and anxiety about cancer, or remorse over not receiving more aggressive treatments [29]. Relying on the CSM, the FCR Cognitive Formulation model [29] posits that the level of FCR varies depending on the cognitive and emotional representations of cancer, including the level of the perceived threat of cancer, which may provoke negative emotional representation (e.g., depression, horror, anger). The cognitive and emotional representations of cancer ultimately inform FCR levels. 

In addition, the Conservation of Resources theory (COR) [30] posits a mechanism by which promotive and protective factors operate to strengthen coping with stressful experiences. When coping with a stressful condition, such as cancer, individuals tend to protect and acquire new resources to handle it [31]. Resources can include objects, states, and conditions that people value (e.g., personal-psychological competence, family relations) [30]. Individuals invest resources for the sake of defending against resource loss, recovering from losses, and gaining resources [32]. The number of resources determines a spiraling process of resource gain or loss, which determines the perceived capacity to handle the situation [33]. Hence, according to the COR theory, women diagnosed with BC who have greater resources may be able to preserve and acquire new resources to cope with BC, which may influence their perceived stress [34] and their FCR levels [29]. Further, having more resources may lead to resource gain and better adaptation over time [34]. Therefore, in this study, we focused on two resources against FCR growth, following a BC diagnosis. One resource pertains to a specific coping ability with cancer, namely coping self-efficacy, while the other refers to a general coping ability, i.e., positive cognitive–emotion regulation. 

### Protective Factors against FCR

One of the personal characteristics that can contribute to lower FCR levels is self-efficacy. Self-efficacy refers to an individual’s perception of their ability to successfully perform actions that can lead to overcoming challenges and achieving goals [35]. Perceived self-efficacy is formed through evaluating one’s behaviors and coping skills in dealing with a certain situation [36,37]. Individuals with high self-efficacy tend to perceive themselves as more capable of dealing with threats [37] such as illness and, therefore, will perceive greater control and adaptive health behaviors [38]. 

Furthermore, specifically, self-efficacy in coping with cancer (or coping self-efficacy) refers to the confidence one has in managing symptoms and emotions related to BC and it includes behaviors directed to benefit the process of recovery [39], such as seeking help, informing and reporting about symptoms, and adhering to follow-ups upon completion of the treatments [40]. Studies have demonstrated that BC patients with higher levels of coping self-efficacy achieve more favorable psychosocial and medical outcomes, such as experiencing fewer side effects, compared to those with lower coping self-efficacy [41]. Consequently, it is possible that BC patients with greater coping self-efficacy perceive their condition as more controllable [26], resulting in lower levels of FCR. 

Consistent evidence indicates a link between higher levels of coping self-efficacy and lower levels of FCR, including in BC patients [21,42]. This finding has been observed in various studies conducted at different stages of the treatment and survivorship journey. For instance, a prospective longitudinal study demonstrated an association between greater coping self-efficacy and reduced FCR levels following cancer treatment completion [21] and among young BC survivors [43]. Greater coping self-efficacy also predicted lower FCR six to 24 months after the completion of the primary treatment [42,44]. In addition, coping self-efficacy mediated the effects of risk factors (e.g., anxiety and younger age) on FCR among survivors who were three to eight years post-diagnosis [40]. Lastly, clinically, in a four-week online intervention study, focusing on BC survivors with moderate to high FCR levels, greater coping self-efficacy predicted a significant decrease in FCR from baseline to post-intervention [45]. 

Moreover, cognitive–emotion regulation has been recognized as a significant predictor of better mental health in oncological patients [46], and thus may also protect against the development of FCR. Cognitive–emotion regulation is a cognitive process by which individuals manage emotionally arousing information, through monitoring, evaluating, and modifying responses to an event [47]. Individuals vary in their capacity to regulate emotions [48] by using thoughts to manage their emotions [49]. Cognitive–emotion regulation includes maladaptive and adaptive strategies. Adaptive cognitive emotion–regulation strategies include positive refocusing, positive reappraisal, putting the issues into perspective, acceptance, and planning [48,49]. These cognitive–emotion regulation strategies result in better well-being and psychological functioning [47]. The CSM [26,27] posits that personal characteristics, such as cognitive–emotion regulation, may affect the way women cope with BC. More specifically, it may impact the way women perceive and evaluate the severity of their illness, which informs their cognitive and emotional representations of cancer [26]. Finally, these representations can eventually mitigate FCR levels [26,29].

Indeed, former studies have demonstrated the correlation between positive cognitive–emotion regulation strategies and a lower FCR. Two clinical studies, including a 12-week emotion regulation group intervention [50] and emotion-focused therapy [51], showed a decrease in FCR levels after treatment. Similarly, a qualitative focus group study found that using acceptance and positive reappraisal were effective in coping with FCR [52]. Additionally, a prospective study that examined FCR levels, before and after radiation therapy, indicated that emotion regulation strategies, including reappraisal, are linked with lower levels of FCR [53]. 

The extant research suggests that coping self-efficacy [40] and positive cognitive–emotion regulation can play a protective role against FCR among BC survivors [53]. However, evidence regarding this association from the crucial times of diagnosis, treatment, and early recovery is lacking. Moreover, the predictive-moderating role of coping self-efficacy and positive cognitive–emotion regulation, as well as the exploration of individual differences in FCR over time, have not yet been studied. These gaps are addressed in this study.

**Figure 1:**
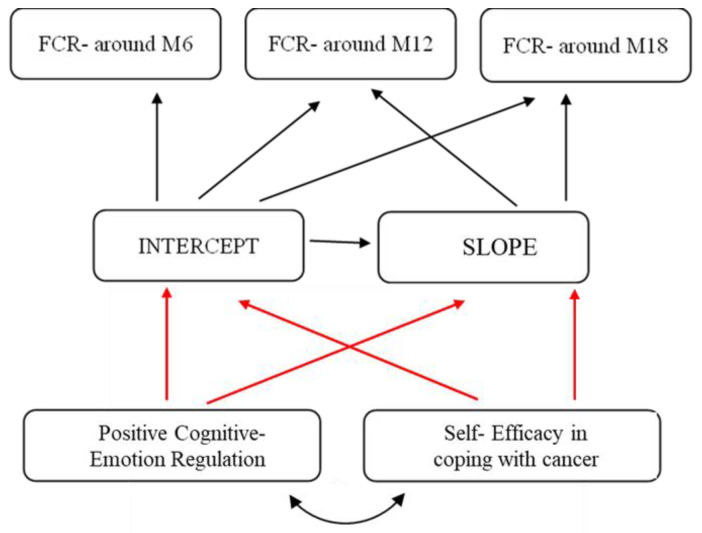
A conceptual model. A latent growth modeling analysis of positive cognitive–emotion regulation and self-efficacy in coping with cancer, predicting the fear of cancer recurrence trajectories. Note. Red darts = represent predicted negative correlations; black darts = represent predicted positive correlations.

Relying on the CSM [26,27] and the COR theory [30], the present study aimed to examine a longitudinal model, among an international sample of women coping with BC, by which two main protective factors would predict FCR and the development of the FCR trajectory. Specifically, we tested whether higher levels of coping self-efficacy and positive cognitive–emotion regulation, assessed at the time of BC diagnosis, would show a steeper decline in FCR levels at six months after diagnosis, and in the development of the FCR trajectory 12 and 18 months after diagnosis (see Figure 1). This period constitutes the approximate timeframe for managing both acute survivorship and the transitional stage of survivorship—meaning, the experience of diagnosis and receiving medical treatment—followed by the end of active treatment and the efforts to readjust to life [54,55]. Identifying individual differences in growth trends of FCR may foster the development of interventions designed to prevent FCR.

Based on the lack of consensus in the literature regarding the change patterns of FCR over time, the first research question aimed to explore whether FCR levels (six months post-diagnosis) predict the trajectory of FCR levels later at 12 and 18 months post-diagnosis. Next, the following hypotheses addressed the two protective factors, such that greater coping self-efficacy measured at the time of diagnosis of BC will predict: (H1.a) a lower level of FCR at six months, and (H1.b) a steeper decline in FCR levels at 12 and 18 months since the diagnosis. Similarly, greater positive cognitive–emotion regulation measured at the time of diagnosis of BC will predict: (H2.a) a lower level of FCR at six months, and (H2.b) a steeper decline in FCR levels at 12 and 18 months since the diagnosis. 

## 2. Materials and Methods

### 2.1. Procedure

Data for the current study were obtained through an international longitudinal re-search project named “Predicting Effective Adaptation to Breast Cancer to Help Women to BOUNCE Back” (BOUNCE). The BOUNCE study was conducted as part of a consortium project that promoted collaboration among countries and European oncology centers. Researchers, doctors, and therapists worked together in a multidisciplinary study, involving a multicultural sample from four oncology centers, each with extensive experience in the holistic treatment of many BC patients. These centers were in Italy, Finland, Israel, and Portugal. Participants enrolled for the study had to be: women between 40 and 70 years of age, with a recent diagnosis of histologically confirmed invasive early or locally advanced operable BC, tumor defined as stage I to III, receiving any type of systemic and local treatment according to local guidelines for BC, and capable of understanding the study protocol and providing informed consent. Women diagnosed with distant metastases, a history of another malignancy within the last five years (except cured basal cell skin carcinoma or in situ carcinoma of the uterine cervix), a history of severe mental, neurologic, or another chronic disease, and pregnant or breast-feeding at the time of recruitment, did not much the study’s criteria. Ethical certifications were approved for the study by the ethical committee of the European Institute of Oncology (IEO; Approval No. R868/IEO916) and through ethical committees at each medical center.

Baseline assessments were conducted following the cancer diagnosis and before receiving medical treatment. The participating women were recruited for the study during their first hospital appointments or through a phone call initiated by a research assistant. A brief description of the study and its goals was presented. Following the signing of an informed consent form, they filled out a battery of self-report questionnaires in their local native language. After the initial assessment, the subsequent phases (six-month, 12-month, and 18-month follow-ups) were carried out by the research assistants from each medical center. At each time point, the participants were given the questionnaires in the form of printed or online surveys using the Noona survey software or the Qualtrics platform. The translation process of the questionnaires was obtained from the original tool developer or conducted by translation experts through a back-translation process. The medical data needed were assembled through the hospitals’ medical record files. 

### 2.2. Participants 

The baseline assessment included 690 women, from whom 574 completed the six-month follow-up, 525 completed the 12-month follow-up, and 494 completed the 18-month follow-up, yielding an 83.18%, 74.4%, and 70% retention rate, respectively. Relevant tests were conducted to examine distinctions between participants who dropped out and participants who completed the study. Women retained at the six-month follow-up differed from the women who dropped out in the factors of country of origin (χ^2^(3, N = 706) = 95.25, *p* = 0.000), income level (χ^2^(1, N = 663) = 6.80, *p* = 0.009), and education level (χ^2^(1, N = 702) = 7.52, *p* = 0.006). Significant differences also emerged at the 12-month follow-up, in the country of origin (χ^2^(3, N = 706) = 98.41, *p* = 0.000), marital status (χ^2^(1, N = 696) = 5.13, *p* < 0.05), income level (χ^2^(1, N = 663) = 5.36, *p* < 0.05), and education level (χ^2^(1, N = 702) = 13.64, *p* = 0.000). Finally, women who completed the 18-month follow up differed from the women who dropped out based on their country of origin (χ^2^(3, N = 706) = 122.96, *p* = 0.000), income level (χ^2^(1, N = 663) = 11.62, *p* = 0.001), and education level (χ^2^(1, N = 702) = 34.03, *p* = 0.000). At all three time points, the women dropping out were more often Italian participants, and with lower income and educational levels. There were no significant differences in age, number of children, and disease stage between participants who dropped out and those who remained. Most relevant, there were no statistical differences in the levels of FCR at six months between participants that completed the study and those who dropped out. 

Finally, the study sample comprised 494 women diagnosed with BC, ranging from tumor stage I to III and receiving BC treatments. Most of the women were diagnosed with stage I (47.9%) and II (41%), and a minority with stage III (11.1%). Participants ranged in age from 40 to 70 years, with an average age of 54.93 years (SD = 8.21). The largest group of participants were from Finland (31.9%), then from Italy (27.5%), Portugal (20.8%), and the least from Israel (19.8%). Majority of the women were married or were involved in an intimate relationship (73.7%), with a mean of 1.95 (SD = 1.46) children. At least 76% reported an average or higher-than-average family income level and majority of participants had earned at least a bachelor’s degree (68.1%). 

### 2.3. Measures

Socio-demographic data. A socio-demographic questionnaire assessed age, number of children, marital status (i.e., 0 = “married, common law partner, engaged”; 1 = “separated, divorced, single, widowed”), income level (i.e., 0 = “very low”; 1 = “average/high”), and education (i.e., 0 = “primary, secondary, high school”; 1 = “bachelor, postgraduate education, vocational non-academic diploma”). 

Fear of cancer recurrence. FCR was assessed using the Fear of Cancer Recurrence Inventory-Short Form (FCRI-SF) [56], a nine-item, self-report measure derived from the full 42-item form, assessing the presence and severity of intrusive thoughts associated with FCR (e.g., “How much time per day do you spend thinking about the possibility of cancer recurrence?”). The items are rated on a five-point Likert scale ranging from 0 (”not at all”) to 4 (”a great deal”). A higher total mean score indicates higher levels of FCR [56]. In the current sample, the reliability coefficients of the scale at all three time points were high, with Cronbach’s α = 0.86, α = 0.85, and α = 0.87, at 6, 12, and 18 months, respectively. 

Self-efficacy in coping with cancer. Self-efficacy in coping with cancer was measured with the Cancer Behavior Inventory-Brief Version (CBI-B) [57], which is a 12-item scale assessing self-efficacy for coping with cancer, including 4 aspects: maintaining independence and a positive attitude, participating in medical care, coping and stress management, and managing effect (e.g., “actively participating in treatment decisions”). The short version was derived from the full 33-item questionnaire (CBI-L) [58]. The items are rated on a 9-point Likert scale, ranging from 1 (“not at all confident”) to 9 (“totally confident”). A higher total mean score indicates greater self-efficacy beliefs for engaging with the resources needed to cope with cancer. Internal consistency for the scale was high (α = 0.89).

Positive cognitive–emotion regulation. The positive cognitive–emotion regulation scale was measured using the Cognitive–Emotion Regulation Questionnaire (CERQ short) [59], an 18-item, self-report measure for positive and negative cognitive–emotion self-regulation strategies. The short version was derived from the full 36-item questionnaire, comprising nine domains of emotion regulation strategies to cope with a stressful situation. The current study focused on 10 items, assessing the positive cognitive–emotion regulation strategies (e.g., “I think that I have to accept that this has happened”). The positive scale assesses putting the issues into perspective, positive refocusing, positive reappraisal, acceptance, and planning. Each domain is represented by two items in the short form. The items are rated on a five-point Likert scale, ranging from 1 (“almost never”) to 5 (“almost always”). A higher mean score indicates higher positive cognitive–emotion regulation [59]. In our data, the reliability coefficient of the CERQ positive subscale was high (Cronbach’s α = 0.81).

### 2.4. Data Analysis

Preliminary analysis was conducted using SPSS version 25. The study hypotheses were tested using Structural Equation Modeling with AMOS, version 25 [60]. We utilized a latent growth model (LGMs) [61,62] to assess the growth trajectory of FCR levels and their predictors. This analysis estimated the change in repeated measures of FCR levels at six, 12, and 18 months after BC diagnosis. It also tested for the associations between the intercept (i.e., the initial level of FCR) and the slope (i.e., indicates the average rate of change of mean FCR) of the FCR levels. The hypotheses regarding the predictors of FCR growth were tested using a conditional model that included coping self-efficacy and positive cognitive–emotion regulation as predictors of the intercept and the slope of FCR. Model fit was evaluated by standard criteria, including a non-significant (*p* > 0.05) chi-squared statistic, a comparative fit index (CFI) and normed fit index (NFI) of more than 0.95, and a root mean square error of approximation (RMSEA) of less than 0.08 [63].

Since the preliminary analysis showed that some demographic variables were associated with missing data in FCR, including education, income, marital status, and country of origin, they were included as covariates in the model. We also included age as a covariate because it was found as a predictor of FCR in other studies [12,64,65]. We controlled for the nominal variable, country of origin, by converting it to three dummy variables and the quantitative variable age. Finally, we used the full information maximum likelihood (FIML) to handle missing data. FIML uses all available information from the observed data in the SEM analyses and is preferable to mean imputation and listwise or pairwise deletion [66]. 

## 3. Results

### 3.1. Preliminary Analysis

The Skewness and Kurtosis values indicated that the variables in the study did not present a significant bias to normal distribution (Skewness varied between −0.83 and 0.14, and Kurtosis between −0.42 and 0.21). The descriptive statistics and zero-order correlations of the study variables are displayed in Table 1. 

The two protective factors against FCR—coping self-efficacy and positive cognitive–emotion regulation—were positively associated. Coping self-efficacy was negatively associated with FCR at all three time points. Positive cognitive–emotion regulation was not linked to FCR levels at any time point. The FCR levels at the three time points were positively associated with each other. 

### 3.2. FCR Change from Six to Eighteen Months after BC Diagnosis 

First, an unconditional LGM was run to assess the change along the trajectory of FCR levels between six, 12, and 18 months after BC diagnosis. The model adequately fit to the data (χ^2^(df = 1)) = 0.140, *p* = 0.71; NFI = 1.00, CFI = 1.00, RMSEA = 0.000). Although baseline levels of FCR presented significant individual differences between participants (b = 0.33; SE = 0.03; Z = 9.94; *p* < 0.001) around a mean level of FCR (b = 1.68; SE = 0.03; Z = 60.27; *p* < 0.001), there were no significant changes over time, as indicated by the non-significant estimate of the slope’s mean (b = −0.40; SE = 0.03; Z = −1.57; *p* = 0.117). In other words, the growth rate of FCR was homogeneous among participants considering the non-significant variance around the mean growth (b = 0.03; SE = 0.06; Z = 0.350; *p* = 0.727). The correlation between the intercept and the slope was not found to be significant (r = 0.035, *p* = 0.300). 

**Figure 2:**
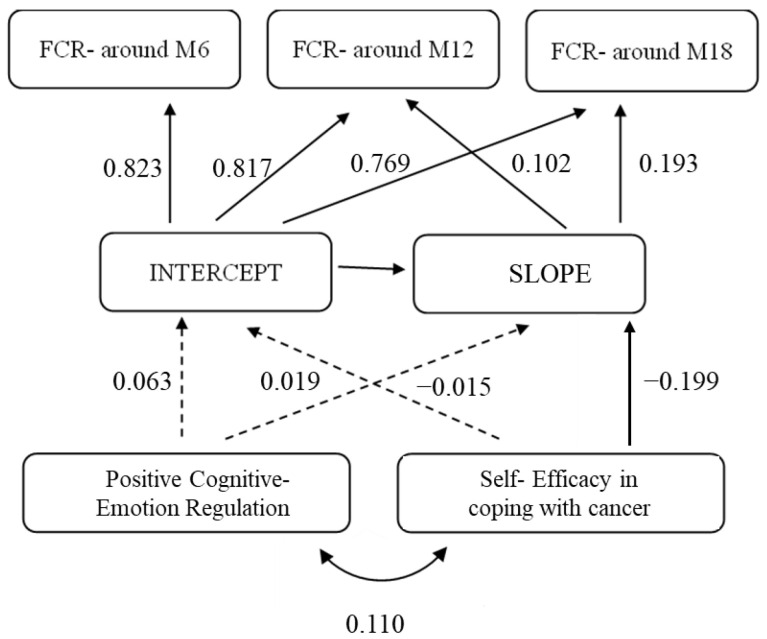
Latent growth modeling: coping self-efficacy predicts initial FCR levels. Note. Full dart = significant correlation; broken dart = non-significant correlation.

Further, a conditional model was used to examine the effect of coping self-efficacy and positive cognitive–emotion regulation on the change in FCR levels (see Figure 2). First, this model was examined with all covariates, including marital status, income level, education, age, and country of origin. However, only age and country of origin emerged as significant predictors of intercept FCR levels; therefore, all other covariates were excluded from the final model. 

The model fit the data very well (χ^2^(df = 9) = 16.42, *p* = 0.06; NFI = 0.99, CFI = 99.05, RMSEA= 0.03). Results showed that coping self-efficacy (at baseline) significantly predicted FCR at six months (β = −0.199; *p* < 0.001), indicating that women reporting greater coping self-efficacy at baseline reported lower levels of FCR after six months. No significant variance was found; thus, coping self-efficacy did not predict the growth rate of FCR later (β = −0.015; *p* = 0.508). In addition, positive cognitive–emotion regulation did not predict either the baseline levels (β = 0.063; *p* = 0.278) or the growth (β = 0.019; *p* = 0.735) of FCR over 18 months.

Finally, younger age was found as a significant covariate to a greater initial FCR level (β = −0.015; *p* < 0.001) but not the growth of FCR (β = 0.002; *p* = 0.526). Additionally, living in Israel (β = 0.338; SE = 0.084; *p* < 0.001) and Finland (β = 0.276; SE = 0.075; *p* < 0.001) were linked to higher FCR levels at baseline; further, living in Israel also predicted a sharper decline in FCR (β = −0.210; SE = 0.082; *p* < 0.05). 

## 4. Discussion

The current study aimed to explore the trajectory of FCR levels between six and 18 months post-diagnosis. Additionally, the objective of this study was to examine two protective factors, namely coping self-efficacy and positive cognitive–emotion regulation, against FCR levels and their trajectories over six, 12, and 18 months following the initial diagnosis of BC. The findings partially supported our hypotheses and have implications for early interventions to improve and mitigate FCR among women with BC.

We identified relatively stable levels of FCR over one year, from six to 18 months after BC diagnosis. Our findings resemble previous studies, showing that FCR levels tend to remain steady over time [15,16,17,18] and have been found to be determined by the baseline levels [18,67]. Still, it should be noted that the FCR trajectory was measured in previous studies using diverse timeframes and intervals compared to the present study (e.g., two months after completing treatment, 18 months after surgery). Thus, it could be that the immutability of FCR in the current results is related to the timeframe of our study, during which women were dealing with intense treatments. The relative stability in FCR might change once survivors move on to a long-term follow-up phase, which includes less frequent visits and communication with the oncological team, as well as decreased family support to address arising fears, in comparison to the active treatment period [18]. The importance of recognizing the changes versus the stability of FCR along the survivorship trajectory is emphasized while concluding that the first six months post-diagnosis represent a window of opportunity to effectively impact the initial levels of FCR, that may not significantly change thereafter.

The current results suggest that the initial levels of FCR may develop through coping self-efficacy; however, it may not further promote a later decrease in FCR. Specifically, a higher coping self-efficacy at the time of diagnosis predicted lower FCR levels after six months. These findings align with previous studies that have linked a higher self-efficacy to a lower FCR [21,40,42,43,44]. Nonetheless, the current study expands the scope of existing literature by examining the effect of coping self-efficacy on FCR among an international sample of women, starting from BC diagnosis and continuing over time.

This study contributes to the theoretical literature by the notion that belief in one’s capacity to manage cancer is crucial for successful coping with FCR [29]. These findings support the Common-Sense Model [27], which posits that the response to BC diagnosis and treatment is a subjective process involving mutual influences between exposure to a health threat and cognitive and emotional representations and responses [27,28]. The results of this study stress the significance of the beliefs of patients in their ability to influence their thoughts, emotions, and behaviors [37], specifically regarding coping with BC and its requirements, such that coping self-efficacy promotes a perception of competence to manage the illness [37], provides a higher sense of control [68,69], and ameliorates cancer-related stress [68,70]. Therefore, a higher coping self-efficacy can lead to a lower FCR [18,71,72,73]. 

Nonetheless, in the current study, greater coping self-efficacy measured at the time of diagnosis of BC did not predict a decline in FCR levels at 12 and 18 months since diagnosis. Even though a woman with coping self-efficacy may invest this resource initially, it has been found that over time, she may need additional and diverse resources [32] to cope with FCR and the demands that arise at each stage of receiving and completing treatment [54,55]. This suggests that as women may lose resources (e.g., coping with FCR), they will strive to protect their remaining resources (e.g., their initial ability of coping self-efficacy), such that managing FCR over time is a dynamic and ongoing demand. In addition to the bio-psychological aspects assessed in the current study, social perspectives such as the crucial role of social support [74] should be considered in the model examining protective factors that could alleviate FCR. The complexity of FCR is further evident in studies among BC survivors, who reported FCR as an intense, difficult, multi-dimensional experience [75], including dealing with feelings of being trapped in insecurity, experiencing suffering alone [76], a lack of control, and fear of death [77]. Thus, it seems that coping with FCR over time requires a wide range of resources. Hence, it is important to further explore protective resources and their ability to address FCR throughout the survivor’s journey.

In contrast to our prediction, we found that greater positive cognitive–emotion regulation did not contribute to the level or reduction of FCR. This unexpected outcome may be explained by the need for specific and unique coping strategies when dealing with a BC diagnosis and FCR. For example, acquiring knowledge about BC [5], utilizing tools to manage uncertainty [78], and practicing mindfulness [79] are important, in addition to general positive cognitive–emotion regulation strategies. Specifically, although enhancing positive cognitive–emotion regulation strategies has been found effective for managing FCR among cancer patients in clinical studies [50,51], this connection is not evident when considering it as an inherent personal tendency. Furthermore, the negative impact of BC on psychological well-being might diminish over time, allowing for greater positive psychological changes to occur in the years following diagnosis [80]. Therefore, while positive cognitive–emotion regulation may not alleviate FCR at the time of receiving a BC diagnosis and undergoing active treatments, its role in later stages of survival warrants long-term assessments.

Finally, we found that younger age and country of origin predict FCR levels. Prior research indicated that younger women expressed higher FCR levels [65], but no change in FCR over time. Understandably, worries regarding health, womanhood, parenting, and death worsen FCR [40], especially at a younger age [65]. 

This international sample has shown some cross-cultural differences. Patients from Israel and Finland reported greater initial FCR levels compared to patients from Portugal. The finding of higher FCR levels in Finland and Israel may be attributed to a combination of the level of diagnosis services, the quality of the medical system, options for effective treatments, and mortality rates [81,82]. However, as the study progressed, patients from Israel exhibited a decline in FCR over time, which can be associated with the high survival rate of BC patients in Israel compared to the OECD [83]. This decline may also be related to the effective universal public free health services that address the needs of cancer patients and their families, providing social security benefits and supportive psychosocial interventions [84]. Perhaps becoming more familiar with the system and receiving comprehensive support may ease patients’ FCR.

### 4.1. Limitations and Future Directions

Several limitations of the current study need to be considered. First, the use of self-report measures can attract potential biases and statistical artifacts (i.e., memory and recall issues, social desirability bias), suggesting caution while interpreting the study’s findings. Hence, future studies should consider varied type of tools for data collection, such as objective measures and behavioral observation measures. Second, the model was examined in a limited timeframe of 18 months since diagnosis, raising the need for further examination of the FCR trajectory, which may persist many years later [85,86]. Third, the study sample represents women with early-stage BC, middle age, and living in European countries; therefore, it might not be able to fully represent the BC and cancer patients’ population. Despite the advantage of an international sample, intercultural differences were found that were not the focus of the article but indicate an important need to examine the differences in cultural influence and its effect on FCR. Further research should replicate the model, in various populations previously linked with FCR, such as different stages of cancer and cancer types, ages, and cultures, where the concepts of coping and a fear of cancer recurrence can differ.

### 4.2. Clinical Implications

Considering the evidence that FCR tends to be stable over the first 18 months post-diagnosis of BC, it is imperative to find effective ways to combat and mitigate this fear as early as possible. Especially during the first six months after diagnosis, it may be crucial to intervene to try to influence the FCR levels. Moreover, our study highlights the centrality of specific cancer coping skills, namely coping self-efficacy, rather than general skills, which can play a protective role against FCR during this time. The results suggest that strengthening coping self-efficacy during the time of diagnosis and the first six months following it may be an effective tool in fostering healthy disease perceptions [37] and a lower FCR [29]. Several interventions have been shown to be effective in improving self-efficacy among general cancer patients [87] and BC patients [41]; however, it is important to adapt them for women managing BC at the time of diagnosis, with a specific focus on FCR management.

In addition, there is a need to search for more relevant and amendable protective factors that can be implemented in prevention and intervention programs to reduce FCR and enhance the well-being of BC patients. Such programs may contribute to better adjustment among BC patients to the disease and its treatments, ultimately leading to reduced FCR [40,43,44].

## 5. Conclusions

The current study examined predictors of FCR and its trajectory. In line with the CSM and COR theory, the study emphasized the stability found in FCR levels over time and the impact of coping self-efficacy on the initial levels of FCR. However, enhanced positive cognitive–emotion regulation did not contribute to the level or reduction of FCR. These findings hold significance as they suggest the importance of specific coping skills for cancer patients, within a critical timeframe to impact the level of FCR, which is likely to impair the quality of life and mental health of BC survivors.

## Figures and Tables

**Table 1:** Means, standard deviations, and zero-order correlations of the study variables.

	1	2	3	4	5	M	SD
1. PCER	-					2.53	0.47
2. CSE	0.11 **	-				7.14	1.18
3. FCR-M6	0.03	−0.32 **	-			1.68	0.69
4. FCR-M12	−0.02	−0.34 **	0.69 **	-		1.65	0.70
5. FCR-M18	0.01	−0.34 **	0.69 **	0.73 **	-	1.64	0.74

Note. ** *p <* 0.01. PCER, positive cognitive–emotion regulation; CSE, coping self-efficacy; FCR, fear of cancer recurrence (M6/M12/M18: six, 12, and 18 months post-diagnosis).

## Data Availability

The anonymized data that support the findings of this study are available upon request from the corresponding author. The data are not publicly available due to privacy and ethical restrictions.
